# Interaction between von Hippel-Lindau Protein and Fatty Acid Synthase Modulates Hypoxia Target Gene Expression

**DOI:** 10.1038/s41598-017-05685-3

**Published:** 2017-08-03

**Authors:** Wendi Sun, Hiroyuki Kato, Shojiro Kitajima, Kian Leong Lee, Katarina Gradin, Takashi Okamoto, Lorenz Poellinger

**Affiliations:** 1 Cancer Science Institute of Singapore, National University of Singapore, Singapore, 117599 Singapore; 2 Nagoya City University School of Medicine, Nagoya, 467-8601 Japan; 3 Department of Cell and Molecular Biology, Karolinska Institutet, SE-171 77 Stockholm, Sweden

## Abstract

Hypoxia-inducible factors (HIFs) play a central role in the transcriptional response to changes in oxygen availability. Stability of HIFs is regulated by multi-step reactions including recognition by the von Hippel-Lindau tumour suppressor protein (pVHL) in association with an E3 ligase complex. Here we show that pVHL physically interacts with fatty acid synthase (FASN), displacing the E3 ubiquitin ligase complex. This results in HIF-α protein stabilization and activation of HIF target genes even in normoxia such as during adipocyte differentiation. 25-hydroxycholesterol (25-OH), an inhibitor of FASN expression, also inhibited HIF target gene expression in cultured cells and in mouse liver. Clinically, FASN is frequently upregulated in a broad variety of cancers and has been reported to have an oncogenic function. We found that upregulation of FASN correlated with induction of many HIF target genes, notably in a malignant subtype of prostate tumours. Therefore, pVHL-FASN interaction plays a regulatory role for HIFs and their target gene expression.

## Introduction

*VHL* was originally identified as a tumour suppressor gene in the hereditary VHL disease^1^ that develops a limited spectrum of tumours such as clear cell renal cell carcinoma (ccRCC), pheochromocytoma and hemangioblastoma^2^. *VHL* mutations were also found in sporadic renal cell carcinoma^3,4^ and account for approximately 50% of sporadic ccRCC cases. At normal oxygen concentrations (normoxia), HIF-α proteins are ubiquitylated by pVHL in association with an E3 ligase complex and degraded by the proteasome^5–8^. The interaction between the pVHL E3 ubiquitin ligase complex and HIF-α proteins is regulated by oxygen-dependent prolyl 4-hydroxylases (PHDs)^9–12^ which show reduced activity in hypoxia, providing a model for cellular oxygen sensing^13^. At lower oxygen concentrations (hypoxia), HIF-α proteins are stabilized and function as canonical DNA-binding transcription factors^14–16^.

HIF-1α and HIF-2α regulate genes involved in a wide range of physiological events (e.g. angiogenesis, metabolisms, cell proliferation, apoptosis, etc.) with distinct spectrum^17,18^. These regulations primarily cope with decreased oxygen consumption and the resulting deprivation of ATP as well as need for glucose uptake^19,20^. HIF-α appear to be also regulated by other physiological regulators such as signal transducers and metabolites^13,21^. From studies using genetically modified mice, HIF-1α mainly regulates glucose metabolism by activating the expression of glycolytic enzymes, whereas HIF-2α regulates fatty acid metabolism by suppressing the expression of enzymes for lipogenesis and β-oxidation^22,23^. Lines of evidence have shown that HIF-1α and HIF-2α play crucial roles in cancer formation, progression and metastasis^18,24^. pVHL has multiple HIF-dependent and also multiple HIF-independent functions such as in senescence and microtubule stability^25,26^. pVHL can also target various proteins through poly-ubiquitylation^27–29^, and is itself subject to post-translational regulation such as sumoylation^30,31^ and folding by the TRiC chaperonin complex^32^. Some of the phenotypes observed in *Vhl*-inactivated mice may be attributed to these less-characterized properties^33^.

In pursue for novel targets or regulators that could account for the unsolved links and bring about insights into pVHL function, we have identified FASN as a pVHL-interacting protein and analyzed the function. Downregulation of FASN efficiently decreased HIF-α protein levels in a manner dependent on pVHL in cultured cells and mice, whereas upregulation of FASN during adipocyte differentiation accompanied by an increase in HIF-α target gene expression. Together with data analysis of gene expression profiles of cancer patient samples, these findings strengthen the notion that HIF functionally interacts with metabolic pathways.

## Results and Discussion

### FASN physically associates with pVHL

**Figure 1:**
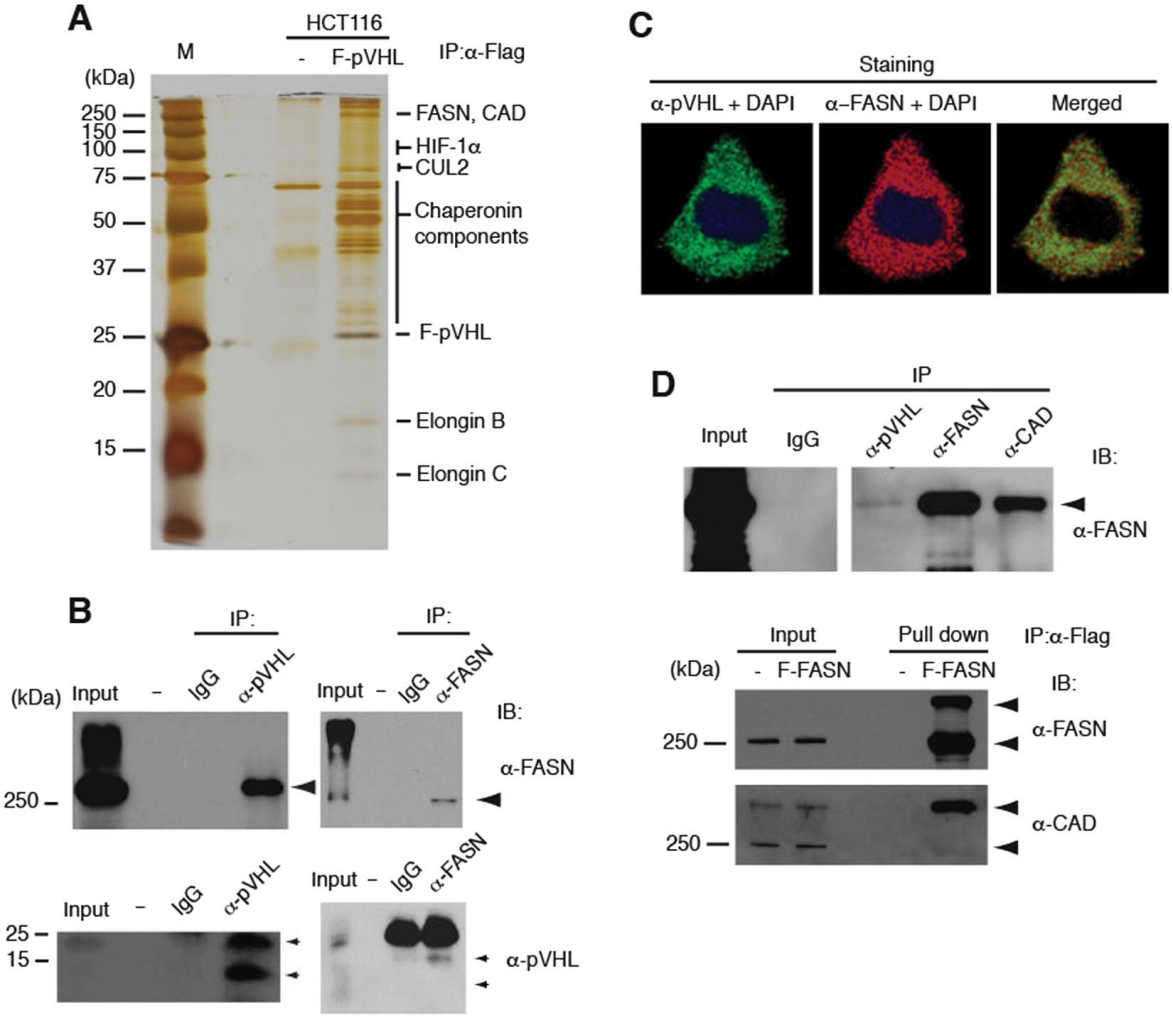
Identification of FASN and CAD as Novel pVHL-associated Proteins. (**A**) Identification of pVHL-associated proteins. Proteins physically associated with Flag-tagged pVHL stably expressed in HCT116 cells were isolated by affinity-precipitation, separated by SDS-PAGE (4–20%) and visualized by silver staining. Identified proteins by LC-MS/MS analysis are indicated on the right (see Table S1 for peptide data of LC-MS/MS analysis). (**B**) Interaction between endogenous pVHL and FASN in HCT116. Large and small arrowheads indicate FASN and pVHL, respectively. pVHL input lane was from long exposure of the same blot. (**C**) Colocalization of pVHL and FASN. Endogenous pVHL and FASN protein in HeLa cells were visualized by immunofluorescence staining. (**D**) Interaction between FASN and CAD. Interaction of endogenous proteins was analyzed using WCEs from HCT116 (upper panel). Flag-tagged FASN stably expressed in HCT116 was tested and found to co-precipitate CAD (lower panel). The experiments were repeated twice (**D**) or more (**A**,**B**,**C**) including mass spectrometric analyses, and the results were reproducible.

pVHL has multiple HIF-dependent and also independent functions occurring through interactions with diverse cellular proteins. To identify novel regulators of pVHL function, we purified cellular proteins physically associated with pVHL. Colorectal HCT116 cancer cells stably expressing full length pVHL with an amino (N)-terminal Flag tag were cultured in the presence of a proteasome inhibitor (MG132), and whole cell extracts (WCEs) were analyzed by affinity purification followed by mass spectrometry (MS). The isolated proteins were separated on 4–20% SDS polyacrylamide gels and visualized by silver staining (Fig. 1A) or zinc-imidazole staining for MS analysis. More than a dozen bands were visualized, some of which correspond to known associated proteins such as Elongin B and C^34^, CUL2^35^ as well as multiple chaperonin components^32^. HIF-1α was also detected in weaker bands as expected. However, other bands corresponding to fatty acid synthase (FASN), carbamoyl-phosphate synthetase 2, aspartate transcarbamylase and dihydroorotase (CAD) represent newly identified major components of pVHL complexes in the 250 kDa region (Fig. 1A and Supplementary Table S1).

Both FASN and CAD are single-polypeptide metabolic enzymes with multiple catalytic domains, and are essential and rate-limiting for fatty acid and pyrimidine biosynthesis respectively^36,37^. The interaction between endogenous pVHL and FASN was confirmed by coimmunoprecipitation assays (Fig. 1B and Supplementary Fig. S1A), and co-localization of the majority of both proteins was observed in HeLa cells by immunofluorescence (IF) staining (Fig. 1C and Supplementary Fig. S1B–D). pVHL was found to bind to the carboxy (C)-terminal half of FASN (Supplementary Fig. S1E). Furthermore, both endogenous and stably expressed Flag-tagged exogenous FASN was also found to bind CAD (Fig. 1D). Upon further investigation of pVHL associated proteins, additional enzymes involved in fatty acid metabolism such as acyl-CoA synthetase 3 and fatty acid coenzyme A ligase 5, were identified in the immunoprecipitated samples (Supplementary Table S1). These results suggest that pVHL is associated with a multi-enzyme complex relevant for fatty acid and pyrimidine metabolism, raising the possibility that these enzymes are co-regulated by pVHL, or pVHL function is in turn regulated by these metabolic components.

**Figure 2:**
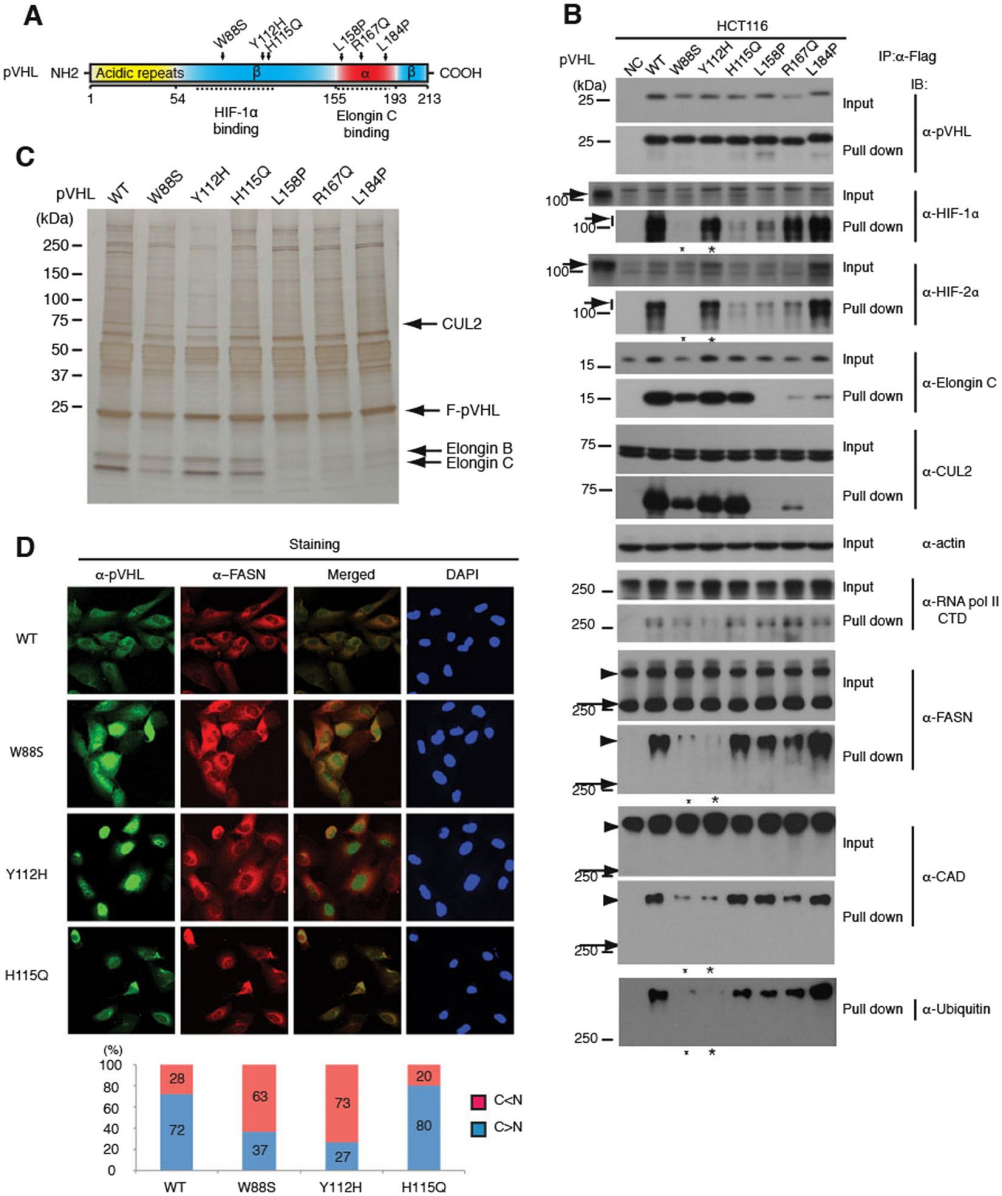
FASN and CAD Binding Specificity of pVHL Mutants Derived from VHL Patients. (**A**) Schematic representation of pVHL domains. Dotted lines indicate the approximate positions of the HIF-1α and Elongin C binding domains. The α and β structural domains are also indicated. (**B**) Effects of pVHL mutations on interactions with E3 ligase components, HIF-α and other interacting proteins including FASN. pVHL mutants were stably expressed by lentiviral vectors in HCT116 cells, and WCEs were tested in precipitation assays. Note that the mutant pVHL-W88S almost completely lost the ability to bind to HIF-1α and HIF-2α, whereas pVHL-Y112H retains this ability almost completely. However, both pVHL-W88S and pVHL-Y112H show significantly reduced binding to FASN and CAD, as indicated by small and large asterisks, respectively. Arrows indicate HIF-1α and HIF-2α bands. Long arrows and arrowheads indicate unmodified and modified forms of FASN, respectively. Protein samples prepared from hypoxia (1% oxygen)-exposed cells were added as positive control in left-end extra lanes. (**C**) The same samples as used in B were analyzed by silver staining. The E3 ligase components appear as major bands as indicated. (**D**) Subcellular localization of stably expressed pVHL mutants in 786-O. pVHL-Y112H and pVHL-W88S showed preferential nuclear localization, whereas pVHL-WT and pVHL-H115Q which both strongly bind to FASN and CAD, showed cytoplasmic localization. Cells showing clear nuclear (C < N) or cytoplasmic (C > N) localization in three microscopic viewing areas were counted as shown in the lower panel. The total counted cell numbers ranged from 35 to 64. The experiments were repeated more than twice, except for the cell localization count, and the results were reproduced.

To assess the functional relevance of the pVHL-FASN interaction, we next examined the effects of pVHL tumour mutations found in VHL patients as depicted in Fig. 2A. Elongin C, which is necessary for E3 ubiquitin ligase activity, and HIF-α proteins directly interact with pVHL domains encompassing the C-terminal and central regions of the protein, respectively. The mutated proteins were first assessed for their abilities to interact with HIF-α proteins and E3 ligase components. WCEs from HCT116 stably expressing Flag-tagged wild-type or mutant pVHL were prepared and tested for interactions by immunoprecipitation and western blot analyses (Fig. 2B). In agreement with previous reports^38,39^, the mutants pVHL-W88S, pVHL-H115Q, and pVHL-L158P showed a dramatic decrease in binding to HIF-1α whereas the mutants pVHL-L158P and pVHL-L184P almost completely lost binding to Elongin C and CUL2. pVHL-R167Q retained binding strongly to HIF-1α and weakly but significantly to HIF-2α and the E3 ligase components, which could reflect weakened HIF-2α degradation activity in pVHL-negative 786-O renal cancer cells (Supplementary Fig. S2). Understanding the underlying mechanism must await further analysis. pVHL-Y112H showed binding to both HIF-α proteins and Elongin C which was indistinguishable from pVHL-WT (Fig. 2B). In control experiments, pVHL-Y112H was able to strongly downregulate HIF-2α levels. This activity was nearly equivalent to that of wild-type pVHL when stably introduced into 786-O (Supplementary Fig. S2).

The specific interactions between mutant pVHLs and E3 ligase components were further confirmed by silver staining (Fig. 2C). The precipitated pVHL mutant proteins were next tested for binding to FASN and CAD as well as a previously reported pVHL-interacting protein^28^ the large subunit of RNA polymerase II (RPB1) (Fig. 2B). Strikingly, pVHL-W88S and pVHL-Y112H showed greatly reduced binding to FASN and CAD, and moderately to RPB1. Intriguingly, we found by IF staining that the majority of pVHL-Y112H and pVHL-W88S proteins were localized in the nucleus, whereas pVHL-WT and pVHL-H115Q were co-localized in the cytoplasm (Fig. 2D). This nuclear localization could be due to loss of binding of pVHL-W88S and pVHL-Y112H to FASN.

### FASN positively regulates HIF-α protein and HIF target genes

**Figure 3:**
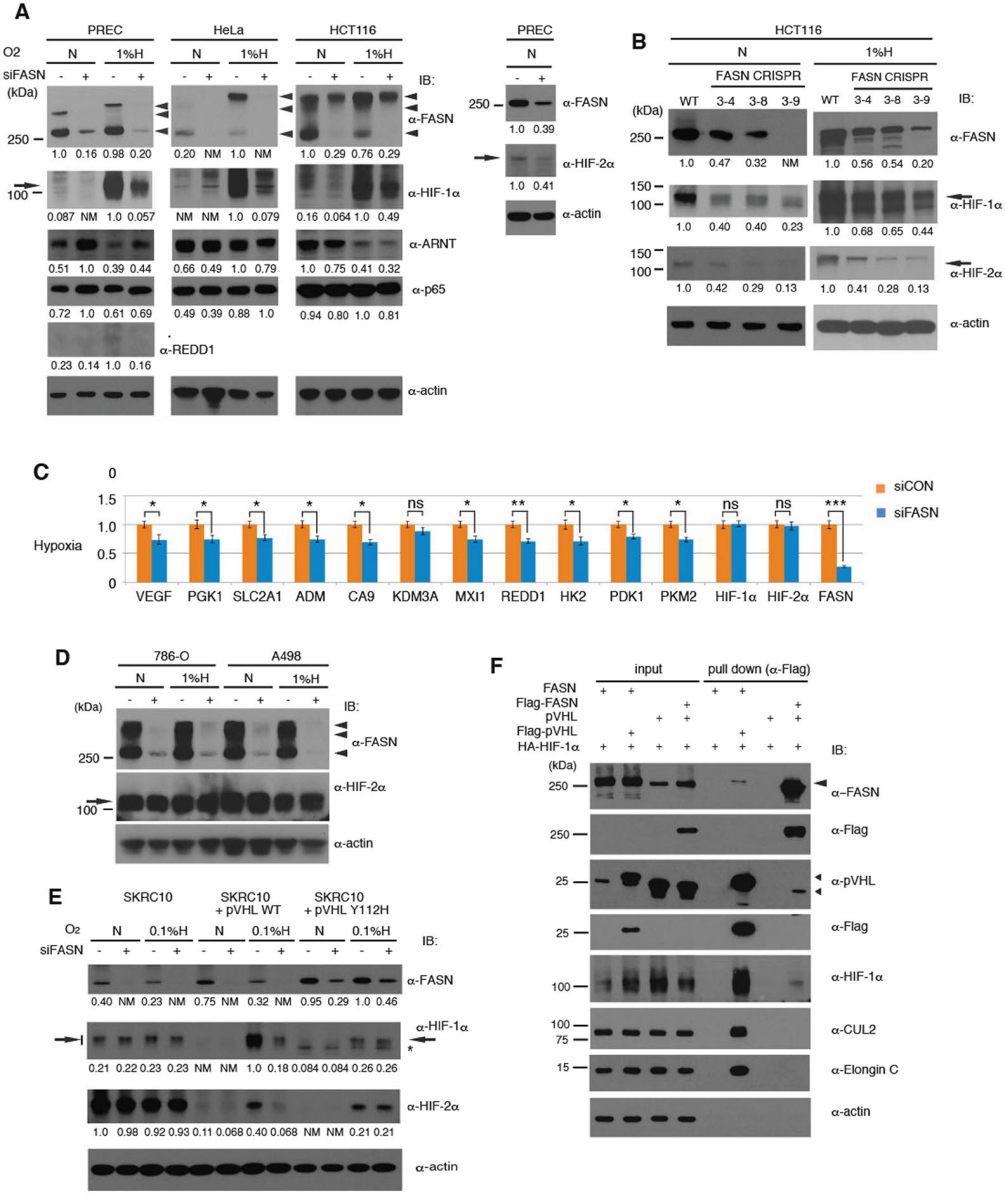
FASN Regulates HIF-α Protein Stability and HIF Target Gene Expression. (**A**) FASN siRNA downregulates HIF-α protein levels. PREC, HeLa and HCT116 cells were treated with *FASN* (+) or control (−) siRNA and harvested after 72 h. Cells were exposed to hypoxia (1% oxygen) or maintained in normoxia (21% oxygen) 4 h before harvesting and subjecting WCEs to western blot analysis. Unmodified (250 kDa) and modified (larger) FASN bands are indicated by large arrowheads, REDD1 by a small arrowhead, and HIF-1α and HIF-2α by arrows. (**B**) Downregulation of HIF-1α and HIF-2α in HCT116 cell clones carrying the *FASN* gene mutagenized using CRISPR-Cas9 (See Supplementary Fig. S3 for genomic DNA information). Protein samples were prepared in normoxia (left column) or hypoxia (right column) for western blot analysis. Asterisk indicates a non-specific band. (**C**) Quantification of HIF-target gene expression by qPCR upon downregulation of FASN in PREC. Samples were prepared from cells treated as described for panel A but with 24 h hypoxia. siRNAs were transfected in triplicates and mRNAs of the HIF target genes were measured in biological triplicates and technical duplicates as a total of 6 samples by qPCR. Values were normalized against the *PPIA* reference gene and control samples. (**D**) Loss of downregulation of HIF-2α levels in ccRCC cells. pVHL-deficient 786-O and A498 cells were tested in FASN siRNA knockdown experiments. (**E**) Restoration of HIF-α regulation by stable expression of exogenous pVHL in SKRC10 cells. pVHL-WT or pVHL-Y112H were introduced into pVHL-deficient SKRC10 ccRCCs using a lentiviral vector carrying a weak promoter and HIF-α protein levels were examined. Specific and non-specific bands are indicated by arrows and an asterisk, respectively. (**F**) FASN and the E3 ligase components are present in the pVHL-containing complexes in a mutually exclusive manner. The indicated constructs were transfected into 293 T cells and WCEs prepared after 48 h were tested for protein-protein interactions by precipitation with an anti-Flag antibody-conjugated resin. Note that FASN failed to precipitate CUL2 and Elongin C, as shown in lanes 6 and 8. Experiments were repeated twice (**D**,**E**,**F**) or more (**A**,**B**), and the results were reproduced. The qPCR experiment (**C**) was performed once with biological triplicates.

To investigate functional importance of the interaction, we tested the effects of siRNA-mediated knockdown of FASN expression on HIF-α protein levels in HeLa, HCT116 and cultured human primary renal epithelial cells (PREC). HIF-1α was highly induced in hypoxia (1% oxygen) and strongly suppressed in PREC and HeLa cells and moderately in HCT116 by reduced FASN expression. In contrast, the control proteins (ARNT, NFkB, p65 and actin) were not downregulated or even slightly upregulated in some cases (Fig. 3A). To confirm this, individual siRNAs were tested on PREC and cultured human primary renal proximal tubule epithelial cells (PRETEC). In a good correlation with FASN suppression, HIF-1α and HIF-2α were clearly downregulated in both normoxia and hypoxia (Supplementary Fig. S3A). This indicates that the downregulation of HIF-α by FASN knockdown was highly specific in various cells. These results suggest that FASN positively regulates HIF-α protein levels.

FASN has been identified as an oncogene in several cancer types^40^, and FASN catalytic activities are believed to at least partially contribute to its oncogenic activity, since the inhibitor cerulenin suppresses tumorigenicity of cancer cells including prostate cancer^41^. We therefore asked whether cerulenin mimics the FASN siRNA suppression effects on HIF-1α protein levels. Exposure to 1% oxygen strongly enhanced HIF-1α stabilization but cerulenin did not induce any significant suppression of these levels in PREC, HeLa and HCT116 cells (Supplementary Fig. S3B), suggesting that the catalytic activity of FASN is not important for this mode of regulation. We next conducted a genome editing experiment using the CRISPR-Cas9 system against the *FASN* gene with targeted nucleotide and altered *FASN* genomic sequences presented in Supplementary Fig. S3AC,D. One cell line (3–9) derived from HCT116 showed strong suppression of FASN expression while two others (3–4 and 3–8) showed moderate reduction (Fig. 3B). In contrast, a potential off-target candidate *KCTD1* was not altered (Supplementary Fig. S3E). In excellent correlation with the downregulation of FASN levels, both HIF-1α and HIF-2α protein levels were significantly lower in the CRISPR-Cas9 edited cells. Moreover, FASN overexpression, albeit moderately, in 293T cells significantly enhanced upregulation of HIF-1α (Supplementary Fig. S3F). To analyze the downstream effects of FASN knockdown on the expression of HIF target genes, quantitative PCR (qPCR) analysis was performed on PREC mRNA samples (Fig. 3C). Numerous known HIF target genes were significantly downregulated upon knockdown of FASN expression. Finally, HIF-1α and HIF-2α mRNA levels were not significantly changed during FASN knockdown, indicating that HIF-α protein levels are regulated by FASN via a post-transcriptional mechanism.

### FASN action on HIF-α requires pVHL

To examine if pVHL is required for the regulation of HIF-α proteins by FASN, we next utilized pVHL-deficient ccRCC cells in FASN knockdown experiments. Since both 786-O and A498 ccRCC cells do not express HIF-1α, HIF-2α levels were analyzed (Fig. 3D). Efficient knockdown of FASN did not affect HIF-2α levels, suggesting that pVHL is likely to be required for FASN to regulate HIF-α protein levels. To directly test this possibility, pVHL was introduced into a pVHL-deficient ccRCC cell line, SKRC10, which expresses both HIF-1α and HIF-2α (Fig. 3E). Lentiviral vectors for expression of moderate levels of pVHL-WT or pVHL-Y112H were employed, since high exogenous pVHL expression strongly suppressed and abrogated hypoxia-induced HIF-α activation in the recipient ccRCC cells and caused severe growth retardation (data not shown). Ectopic expression of pVHL-WT and pVHL-Y112H was able to suppress HIF-1α and HIF-2α levels in normoxia. However, only pVHL-WT permitted the downregulation of HIF-α by FASN siRNA, as seen in hypoxia more prominently (Fig. 3E). Strikingly, upon downregulation of pVHL, HIF-α protein levels were further increased at 1% oxygen concentration (Supplementary Fig. S3G), suggesting that pVHL is still functional under the condition. Thus, taken together, the FASN-pVHL interaction is required for regulation of HIF-α protein levels.

### Complex formation of FASN and pVHL excludes the E3 ligase components

To further understand the molecular mechanism underlying this mode of regulation by FASN, we next examined the composition of FASN protein complexes. Pull down of pVHL precipitated both FASN and the E3 ligase components, whereas pull down of FASN precipitated pVHL but not the E3 ligase components (Fig. 3F, compare lanes 6 and 8). These results suggest that FASN competes with the E3-ligase components for binding to pVHL and consequently renders pVHL unable to poly-ubiquitylate HIF-α proteins for degradation (Supplementary Fig. S3H). Whether FASN directly interacts with and retains pVHL in the cytoplasm remains a subject of furture study, since an indirect interaction could also affect pVHL function. In agreement with this model, introduction of pVHL into 786-O cells did not cause downregulation of FASN, however a slight enhancement of FASN modification was observed (Supplementary Fig. S3I). We detected ubiquitylated FASN in a higher molecular weight range in the precipitation and western analysis using antibodies against ubiquitin in conjunction with the FASN de-ubiquitylating enzyme USP2 (Supplementary Fig. S3J). Nevertheless, these ubiquitin modifications did not lead to proteasome-mediated degradation of FASN.

### A negative regulatory compound of FASN decreases HIF-α protein levels

**Figure 4:**
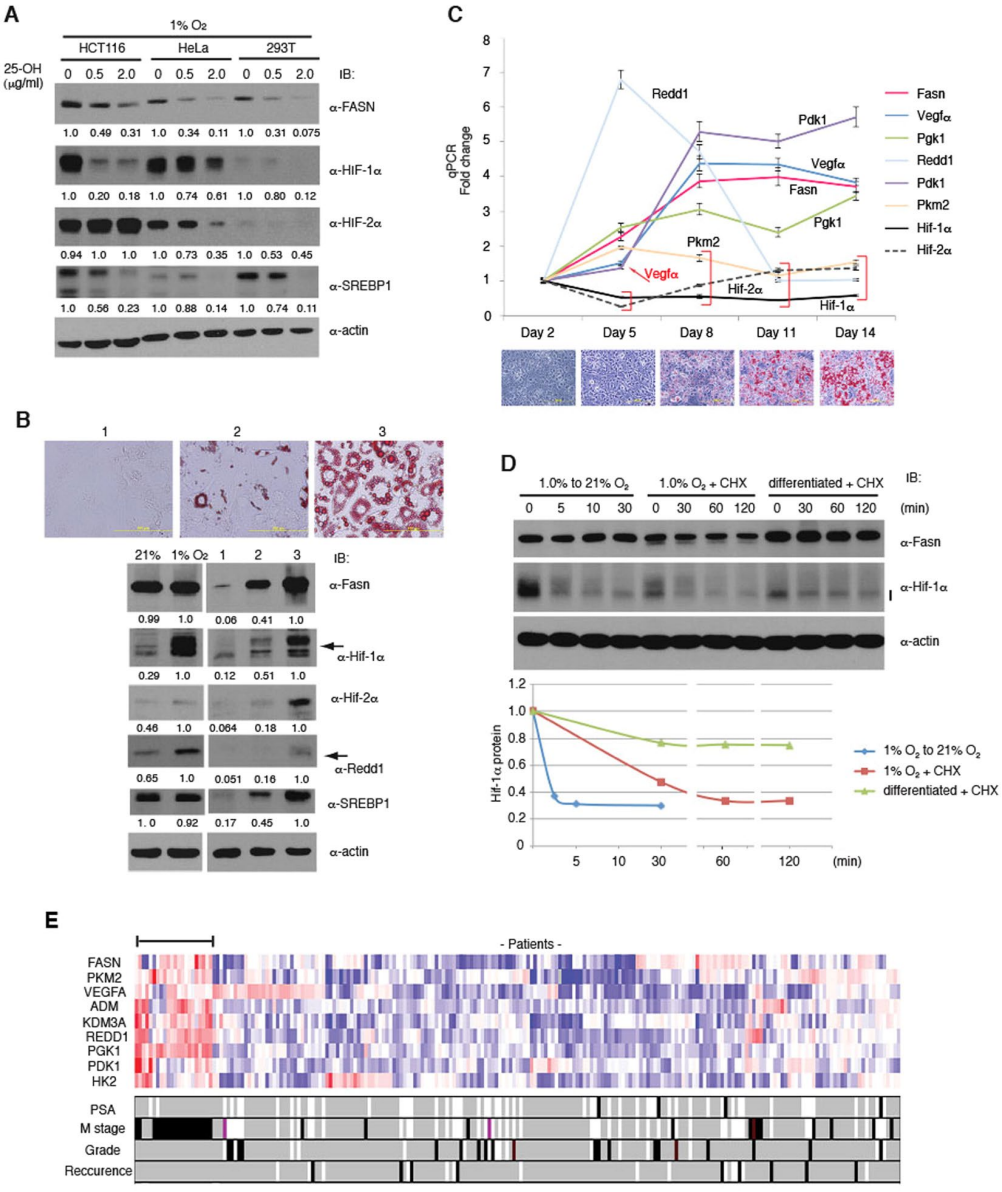
Correlation between HIF-α, FASN Protein Levels and HIF Target Gene Expression in Adipogenesis and Prostate Cancer. (**A**) Downregulation of HIF-α protein levels by 25-OH treatment. HCT116, HeLa and 293 T cells were treated with 25-OH for 72 h and incubated for the last 4 h in 1% oxygen. HIF-α levels were analyzed by western blotting. (**B**) Hif-α activation during differentiation of 3T3-L1 cells into adipocytes. Confluent 3T3-L1 cells were cultured for 14 days in regular medium (1), or differentiation medium in the presence of insulin alone (2) or with insulin, dexamethasone, and 3-isobutyl-1-methylxanthine (3). Fasn, Hif-α and Redd1 expression was analyzed by western blotting. Lipid accumulation was monitored by staining with Oil-Red O. (**C**) Time-dependent changes of Hif target gene expression during 3T3-L1 differentiation. Inducers of differentiation were added at Day 2. mRNAs of the Hif target genes were measured by qPCR in biological triplicates and technical duplicates as a total of 6 samples and normalized against the reference gene *Tbp* and Day 2 samples. Data plots with p-value larger than 0.01 were indicated by red brackets and a red arrow additionally showing Vegfα at Day 2. Lipid staining results with Oil-Red O are shown under the graph for each time point. (**D**) Stability of Hif-1α protein. Stability was measured after oxygen concentration shift (1.0% to 21% oxygen) for undifferentiated cells, cycloheximide (CHX) addition for undifferentiated cells in hypoxia, or cycloheximide addition for differentiated cells in normoxia. (**E**) Gene expression profiles of prostate cancer patients obtained from IST Online (MediSapiens) was analyzed, and mRNA levels of several HIF target genes were examined in correlation with FASN levels. Patient samples co-overexpressed with *FASN* are indicated with a horizontal bar. M stage: metastasis, PSA: prostate-specific antigen. Experiments were repeated twice (**A**,**B**,**D**), and the results were reproduced. The qPCR experiment (**C**) was performed once with biological triplicates.

*FASN* expression is to a large extent driven by the sterol regulatory element-binding protein 1 (SREBP1)^42^. The natural cholesterol metabolite, 25-hydroxycholesterol (25-OH), inhibits proteolytic cleavage of SREBP1 and its translocation into the nucleus in its transcriptionally active form^43^ and thus inhibits target gene transactivation. 25-OH treatment resulted in decreased expression of FASN and processed forms of SREBP1 in HCT116, HeLa and 293 T cells in hypoxia (1% oxygen, Fig. 4A). Concomitantly, HIF-1α and HIF-2α protein levels were also downregulated with some cell type difference (Fig. 4A). In mice, systemic delivery of 25-OH by tail vein injection at low or medium dosage resulted in a significant decrease in mRNA expression of *Fasn* and the Hif target gene *Vegfa* in the liver (Supplementary Fig. S4A), validating the *in vivo* link between Fasn expression and Hif protein levels and activity.

### Positive correlation between FASN and HIF-α expression in physiological contexts

To address the physiological regulation of HIF-α by FASN, we have utilized the system that mouse 3T3-L1 cells differentiate into adipocytes upon administration of the inducers dexamethasone, insulin and 3-isobutyl-1-methylxanthine. During the differentiation, *Fasn* expression is highly induced and as a consequence neutral triglycerides accumulate in lipid droplets^44^. To monitor the extent of the differentiation, Oil-Red O was used which preferentially stains triglycerides strongly. Upon examination (Fig. 4B, upper panel), protein levels of Hif-1α, Hif-2α, and the Hif target Redd1 were found to be highly elevated in the differentiated cells under normoxic conditions (Fig. 4B, lower panel). Expression of a number of Hif target genes was analyzed by qPCR at several time points during adipocyte differentiation in normoxia. Importantly, induced expression of the Hif target genes including *Vegfa*, *Pgk1* and *Pdk1* also correlated with increased levels of *Fasn* (Fig. 4C). Most of the target genes demonstrated an increase at the early phase of differentiation, suggesting that Hif-α activation is a direct consequence of Fasn activation. Interestingly, Hif-1α and Hif-2α mRNAs were not activated (Fig. 4C), which is also consistent with our model that Hif-α protein levels are regulated by Fasn via a post-transcriptional mechanism. To further prove this, we measured Hif-1α protein stability in the 3T3-L1 cells (Fig. 4D). Hif-1α is rapidly degraded in normoxia (half life <5 min), stabilized in hypoxia (half life ~30 min) and strongly stabilized in differentiated cells (half life >30 min).

FASN is highly expressed in numerous cancer types and has been implicated to exert an oncogenic function^40,41^. In prostate cancer FASN has been considered both as a tumour marker and a therapeutic target^40^. This prompted us to determine if there is a correlation in expression between *FASN* and HIF target genes in the clinical context. In agreement with our findings, there was a prominent correlation between *FASN* expression and that of many classical HIF target genes. *PKM2*, *VEGFA*, *ADM*, *KDM3A*, *REDD1*, *PGK1*, *PDK1*, and *HK2* were co-overexpressed in tumours with a malignant phenotype that have metastasis to distant regions (M stage, Fig. 4E). Furthermore, a significant correlation between *FASN* and HIF target gene expression was also found in gastric cancer (Supplementary Fig. S4B). Therefore, these data indicate a concordance between *FASN* and HIF target gene expression in tumour malignancy.

Our finding that FASN regulates HIF function raises the question as to whether HIFs would reciprocally regulate FASN. Genetic inactivation of the *Vhl* gene in mouse liver causes severe steatosis^45^ and shows suppressed expression of genes involved in both fatty acid synthesis and β-oxidation including Fasn^22^. Suppressed *Fasn* expression was reversed by further disruption of the *Hif-2α* but not the *Hif-1α* locus, suggesting that Hif-2α is a major negative regulator of *Fasn*^22^. We therefore addressed the question as to whether HIFs can downregulate FASN in our experimental system. A PHD inhibitor, desferoxamine, did not significantly downregulate FASN in PREC, HCT116 and HepG2 cells, although HIF-1α and HIF-2α protein levels were highly induced (Supplementary Fig. S4C). Moreover, stable overexpression of exogenous HIF-1α or HIF-2α did not change FASN levels in HCT116 and HepG2 (Supplementary Fig. S4D,E). Thus, lack of negative regulation of FASN by HIF-α enables constitutive co-overexpression of both FASN and HIF target genes in cancer cells, which indicates a compatible mechanism of oncogenesis. However, in the normal liver, the negative feedback loop may constitute an important physiological regulatory circuit.

Our data demonstrate that the metabolic enzyme, FASN, regulates HIF protein stability via a physical interaction with pVHL and showed the unexpected link of the lipid metabolism with HIF target gene expression, defining a metabolic switch for activation of the cellular hypoxic response pathway that is independent of oxygen. This mechanism also provides a molecular explanation for the observed increase in HIF-1α levels upon overexpression of MYC which results in significant upregulation of FASN and CAD protein levels^46^. Unlike in normal mouse liver, FASN was not downregulated by HIF-α in human cancer cells, and therefore co-overexpression of HIF-α and FASN allows the maintenance of the malignant state. Whereas numerous metabolism-related genes are regulated by HIFs and the transcriptional activity of HIF-1α is modulated by SIRT6^47^ and the glycolytic enzyme pyruvate kinase isoform M2^48^, the role of metabolic enzymes acting immediately upstream of HIFs has rarely been investigated. The present data establish that pVHL function is directly regulated by interaction with FASN, which could lead to future therapeutic application.

## Methods

### Plasmids

FASN, HIF-1α and HIF-2α plasmids were obtained from the Kazusa DNA Research Institute (Chiba, Japan). Full length human cDNAs for wild-type or mutant pVHL (213 amino acids), HIF-1α, and HIF-2α were inserted into pCSGW^49^, CSII-CMV-MCS-IRES2-Venus (RIKEN) or a derivative vector carrying a minimal HIV promoter with TATA and Sp1 binding sequences. Human *FASN* cDNA was inserted into CSII-CMV-MCS-IRES2-Venus. The *VHL* cDNA sequence was mutagenized using the QuickChange II XL Site-directed Mutagenesis Kit (Stratagene) according to the manufacturer’s instructions.

### Immunoprecipitation

WCEs were prepared by lysing cell pellets in M-PER buffer (Thermo Scientific) containing protease inhibitor cocktail cOmplete (Roche) and 10 µM MG132 prior to immunoprecipitation experiments. Extracts were incubated overnight with anti-Flag M2 affinity agarose or antibody-conjugated Protein-G Sepharose beads. The beads were washed twice with washing buffer (50 mM Tris pH 8.0, 150 mM NaCl, 10 mM NaF, 1 mM EDTA, 1% Triton X-100, 0.2% Sarkosyl, 10% Glycerol) and then twice with buffer D-300 (20 mM Tris-HCl pH 8.0, 300 mM NaCl, 0.5 mM EDTA, 10% Glycerol, 0.5% NP40). Treatment with 2 units/50 μl of DNase I (New England Biolabs) for 30 min at 30 °C did not change the binding properties of identified proteins. Immunoprecipitated proteins were eluted by incubating with buffer D-300 containing 250 μg/ml Flag peptide (for Flag-tagged proteins) or with 1% SDS (for endogenous proteins) and detected by western blot analysis.

### Mass spectrometry analysis

Proteins were resolved by SDS-PAGE and stained with Coomassie Brilliant Blue R-250 or zinc-imidazole. Protein bands were excised for identification by mass spectrometry (ProtTech, PA). Proteins identified with high scores (>30% of relative identified peptide composition within a sample) were taken into consideration and common contaminants were excluded. Detailed data is shown in Supplementary Table 1.

### Immunofluorescence (IF)

Staining of cells was performed by fixation with 4% formaldehyde for 10 min, permeabilization with PBS containing 0.1% NP40 for 15 min, blocking with PBS supplemented with 0.3% Triton X-100 and 5% BSA for 30 min and incubating with primary antibody in PBS with 0.3% Triton X-100 and 1% BSA for 2 h at room temperature. This was followed by incubation with a secondary antibody (Alexa Fluor® 488 or 568, Invitrogen) and mounting with Prolong® Gold antifade reagent (Invitrogen). Photomicrographs were taken on the Nikon A1R confocal microscope with 20×, 40× or 60× objectives.

### Western blot analysis, silver staining and zinc-imidazole staining

Western blot analysis was performed with ECL or ECL prime reagents (GE Healthcare). To analyze high molecular weight proteins such as FASN, samples were applied on 6% polyacrylamide gels and transferred for 6 h at 40 V in a buffer tank to ensure efficient transfer. Specific bands were quantified using Image J and normalized against actin. Relative values were adjusted to 1.0 for the most abundant bands with NM (not measurable) indicating relative values of <0.05. Silver staining was performed with SilverQuest (Invitrogen). For mass spectrometry analysis, protein bands were stained with either Coomassie Brilliant Blue R-250 or zinc-imidazole (E-Zinc Reversible Stain Kit, Thermo Scientific).

### Antibodies

Antibodies used for western blot analysis are as follows: HIF-1α (GTX127309) from GeneTex; HIF-1α (NB100-479) and HIF-2α (NB100-122) from Novus Biologicals; HIF-2α (#7096), pVHL (#2738), FASN (#3180) and ubiquitin (#3936) from Cell Signaling; Elongin C (sc-135895), CUL2 (sc-166506), ARNT (sc-5580), SREBP1 (sc-366), and normal mouse and rabbit IgG (sc-2025 and sc-2027 respectively) from Santa Cruz Biotechnology; CAD (ab40800), RNA polymerase II large subunit CTD (ab5408), and CA9 (ab107257) from Abcam; β-actin (A5441) and anti-Flag M2 (F1804) from Sigma-Aldrich; p65 (610869) from Becton Dickinson (BD) and REDD1 (A500-001A) from Bethyl Laboratories. For immunofluorescence, antibodies against pVHL (BD, 556347) or FASN (#3180) were used. For immunoprecipitation, antibodies against pVHL (BD, 556347), FASN (Santa Cruz Biotechnology, sc-55580) or CAD (Abcam, ab40800) were used.

### USP2 deubiquitylation assay

Poly-ubiquitylated FASN substrate was isolated from WCEs of HCT116 cells stably expressing F-pVHL-WT by pull down and incubated with purified *E. coli*-expressed His-tagged human USP2 for 30 min at 37 °C in the absence or presence of 2.5 μg ubiquitin aldehyde (Santa Cruz Biotechnology, sc-4316) in a 30 μl reaction.

### Knockdown by siRNA and expression by lentiviral vector

siRNAs to human FASN (Dharmacon, ON-TARGETplus SMART pool: 003954-00-0005; ON-TARGETplus set of 4: J-003954-11~14) were transfected with DharmaFECT and cells were harvested after 72 h. Lentiviruses for expressing cDNA (CSII-CMV-MCS-IRES2-venus, RIKEN) were prepared as previously described^50^. Briefly, CSII-CMV-MCS-IRES2-Venus-derivatives were co-transfected with pMDLg/pRRE, pRSV-Rev and pMD2.G into 293 T cells with Lipofectamine 2000 (Invitrogen). Supernatants were harvested on the 2^nd^ and 3^rd^ days and concentrated by centrifugation for 60 min at 12,000 g after passing through a 0.4 μm filter^51^. The suspended virus solution was used for infection in the presence of 5–10 μg/ml Sequa-brene (Sigma-Aldrich). Lentivirus experiments were approved by the Genetic Modification Advisory Committee (GMAC) of the National University of Singapore.

### CRISPR-Cas9 gene editing

Genomic mutagenesis of the *FASN* gene using the CRISPR-Cas9 method was conducted with a lentiviral vector (lentiCRISPR v2) as described^52^. The lentiCRISPR v2 was a gift from Dr. Feng Zhang (Addgene plasmid #52961). The targeted sequence locates to the +97 to +117 region downstream of the initiator ATG in the 2nd exon of *FASN*.

### Treatment with 25-OH

Cells were cultured for 2 days in DMEM supplemented with charcoal-treated FBS and stimulated with 0, 0.5 or 2.0 μg/ml 25-OH for 48 h. In mouse experiments, four male mice (C57BL/6 J) were used for each of control, low or medium doses of 25-OH (0, 2.5 or 10 mg/kg) and were injected twice with 5 ml/kg (25-OH in 20% ethanol per weight) at Days 0 and 1. Livers were isolated at Day 4 for qPCR. Experiments except for mRNA isolation and qPCR were performed in UNITECH (Kashiwa, Chiba, JAPAN). The animal experiments were performed according to the guidelines of the Animal Ethics Committee of UNITECH. The guidelines have been inspected and approved by the Japanese Ministry of Education, Culture, Sports, Science and Technology (MEXT), under the following regulations.

–Act on Welfare and Management of Animals (Act No. 105 of October 1, 1973) Chapter I General Provisions.

–Fundamental Guidelines for Proper Conduct of Animal Experiment and Related Activities in Academic Research Institutions under the jurisdiction of MEXT (Notice, No. 71, 2006).

–Guidelines for Proper Conduct of Animal Experiments (June 1, 2006 Science Council of Japan).

### Cell culture and differentiation of 3T3-L1 cells

All cell lines were cultured in DMEM supplemented with 10% FBS, 2 mM glutamine, 100 U/ml penicillin and 100 μg/ml streptomycin (Gibco) except for primary renal mixed epithelial cells (PREC) (ATCC #PCS-400-012, Lot 58488854) and the human primary renal proximal tubule epithelial cells (PRETEC) (ATCC #PCS-400-010, Lot 63010943), that were cultured in specialized ATCC media (PCS-400-030 and PCS-400-040) and used for 3-4 passages. In hypoxia treatment experiments, cells were incubated in an INVIVO2 400 hypoxia workstation (Ruskinn) under 1% O_2_ or 5% CO_2_ at 37 °C unless otherwise specified. 3T3-L1 cells (ATCC, CL-173) were cultured in DMEM and differentiated with insulin, dexamethasone, and 3-isobutyl-1-methylxanthine (Sigma-Aldrich, I-6634, D-4902, and I-5879 respectively), and stained with Oil Red O (Sigma-Aldrich, O-0625) as previously reported^44^. MG132 (C-2211) and 25-hydroxycholesterol (H1015) were purchased from Sigma-Aldrich. Cerulenin (sc-200827) was purchased from Santa Cruz Biotechnology.

### qRT-PCR

Total RNA extraction was performed using the RNeasy Mini Kit (Qiagen) according to the manufacturer’s instructions. RNA concentration was determined with the Nanodrop ND-1000 spectrophotometer and standardized. cDNA synthesis was conducted using the iScript cDNA Synthesis Kit (Bio-Rad, 170-8891) according to the manufacturer’s protocol. cDNA samples were amplified using the KAPA SYBR FAST qPCR Kit (Kapa Biosystems, KK4602), in 384-well plates and quantified by the 7900HT Fast Real-Time PCR system (Applied Biosystems). The ΔΔC_T_ method was used to calculate gene expression levels. For human cell line samples, gene expression was normalized to *PPIA*; for mouse cell lines and tissue samples, gene expression was normalized to Ppia or Tbp. Normalized gene expression was further calculated against the control samples to show the fold changes. Primer sequences are shown in Supplementary Table 2.

### Analysis of gene expression in cancer patients

Gene expression profiles were obtained from the public database IST Online (MediSapiens). Expression levels are indicated in the heatmap as red for upregulation and blue for downregulation. The PSA marker is an indicator which frequently becomes elevated in prostate cancer, and denoted as white, black and gray for elevated, normal expression levels and no data, respectively. M staging is used for the evaluation of metastasis to distant locations, and denoted as white, black and gray for M0 stage, M1 stage and no data. Grade is for histological grading, and denoted as white, black, red and gray for G2, G3, G4 and no data. Recurrence of the disease is shown as white, black and gray for no, yes and no data.

### Statistics

Unpaired two-tailed Student’s t-tests were utilized to calculate p-values (*: 0.01 < p < 0.05, **: 0.001 < p < 0.01 and ***: p < 0.001). Data are represented as mean ± SEM after normalization.

## Electronic supplementary material

Interaction between von Hippel-Lindau Protein and Fatty Acid Synthase Modulates Hypoxia Target Gene Expression
