# Author Correction: Interaction between von Hippel-Lindau Protein and Fatty Acid Synthase Modulates Hypoxia Target Gene Expression

**DOI:** 10.1038/s41598-018-29095-1

**Published:** 2018-07-20

**Authors:** Wendi Sun, Hiroyuki Kato, Shojiro Kitajima, Kian Leong Lee, Katarina Gradin, Takashi Okamoto, Lorenz Poellinger

**Affiliations:** 1 Cancer Science Institute of Singapore, National University of Singapore, Singapore, 117599 Singapore; 2 Nagoya City University School of Medicine, Nagoya, 467-8601 Japan; 3 Department of Cell and Molecular Biology, Karolinska Institutet, SE-171 77 Stockholm, Sweden

Correction to: *Scientific Reports* 10.1038/s41598-017-05685-3, published online 03 August 2017

The original version of this Article contained a typographical error in the spelling of the author Lorenz Poellinger, which was incorrectly given as Lorenz Poelllinger. This has now been corrected in the PDF and HTML versions of the Article, and in the accompanying Supplementary Information file.

