# Supplementary material for: Interaction between von Hippel-Lindau Protein and Fatty Acid Synthase Modulates Hypoxia Target Gene Expression
Source: Sci Rep. 2017 Aug 3;7:7190. doi: 10.1038/s41598-017-05685-3 (PMC5543055; doi:10.1038/s41598-017-05685-3)
Supplement: Supplementary file 1 — Interaction between von Hippel-Lindau Protein and Fatty Acid Synthase Modulates Hypoxia Target Gene Expression [file 41598_2017_5685_MOESM1_ESM.pdf]

Interaction between von Hippel-Lindau Protein and Fatty Acid Synthase Modulates Hypoxia Target Gene Expression

Wendi Sun[1,4], **Hiroyuki Kato**[1,2,4]*, **Shojiro Kitajima**[1], **Kian Leong Lee**[1], **Katarina Gradin**[3], **Takashi Okamoto**[2], and **Lorenz Poellinger**[1,3]**

[1]Cancer Science Institute of Singapore, National University of Singapore, Singapore 117599, [2]Nagoya City University School of Medicine, Nagoya 467-8601, Japan, and [3]Department of Cell and Molecular Biology, Karolinska Institutet, SE-171 77 Stockholm, Sweden.

[4] Wendi Sun and Hiroyuki Kato contributed equally to this work.
*Corresponding author. E-mail: csihk@nus.edu.sg or csihkato@gmail.com (H. Kato); Phone: +65-6516-1115
** Deceased

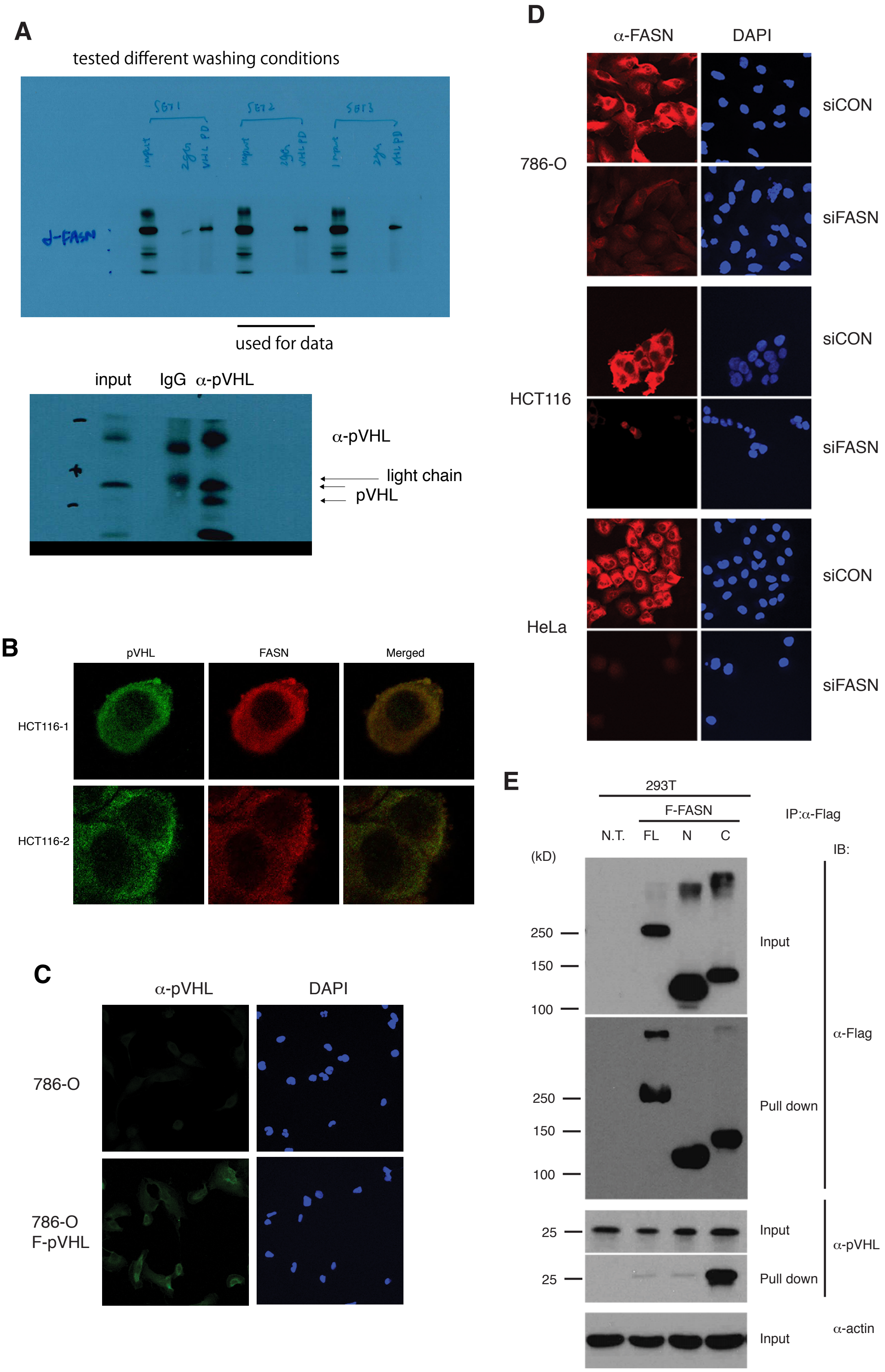

Supplementary Figure 1. pVHL binds to the C-terminal half of FASN. (A) Photos of original films used for Figure 1B. (**B**) Localization of pVHL and FASN in HCT116. (C) Validation of pVHL antibody. 786-O cells stably expressing exogenous F-pVHL were stained with an anti-pVHL antibody (green) and DAPI (blue). Pictures were obtained with the same exposure time. (**D**) Validation of FASN antibody. 786-O, HCT116 and HeLa cells were knocked down with control (siCON) or FASN-targeting siRNA (siFASN) and stained with an anti-FASN antibody (red). These antibodies were used in Fig. 1C. (**E**) Plasmids expressing full length, N-terminal or C-terminal fragments of FASN were transiently co-transfected with a pVHL expressing plasmid into 293T cells, and binding was tested by pull down with anti-Flag resin and western blot analysis.

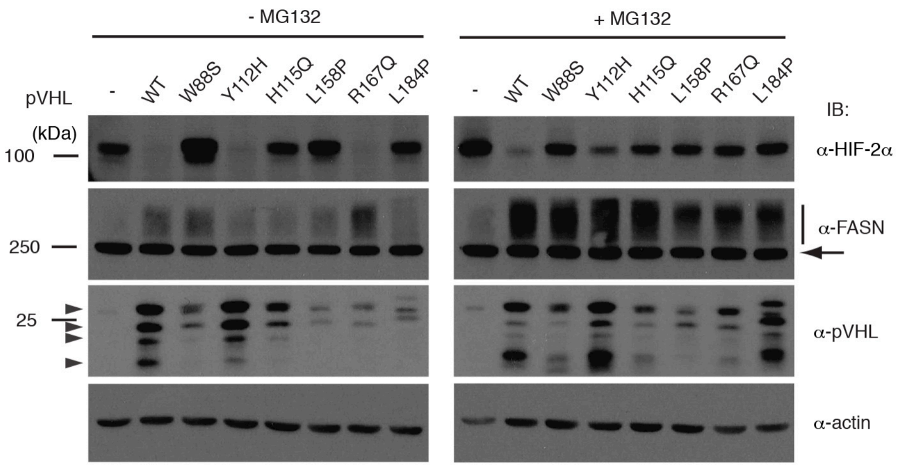

Supplementary Figure 2. Activity of pVHL mutants in downregulation of HIF-2α protein levels in 786-O cells. 786-O cells stably expressing wild-type or pVHL mutants as indicated were cultured in the absence or presence of MG132. HIF-2α levels in WCEs were analyzed by western blotting. Note that pVHL-WT and pVHL-Y112H strongly suppressed HIF-2α protein levels even in the presence of MG132 where ubiquitylation activity was partially blocked. R167Q has strong suppression activity only in the absence of MG132, which could mean an intermediate activity. Flag-tagged pVHL proteins appear as multiple isoforms at 26, 20, 18 and 16 kDa (arrowheads). Unmodified and modified FASN are indicated by arrow and vertical line.

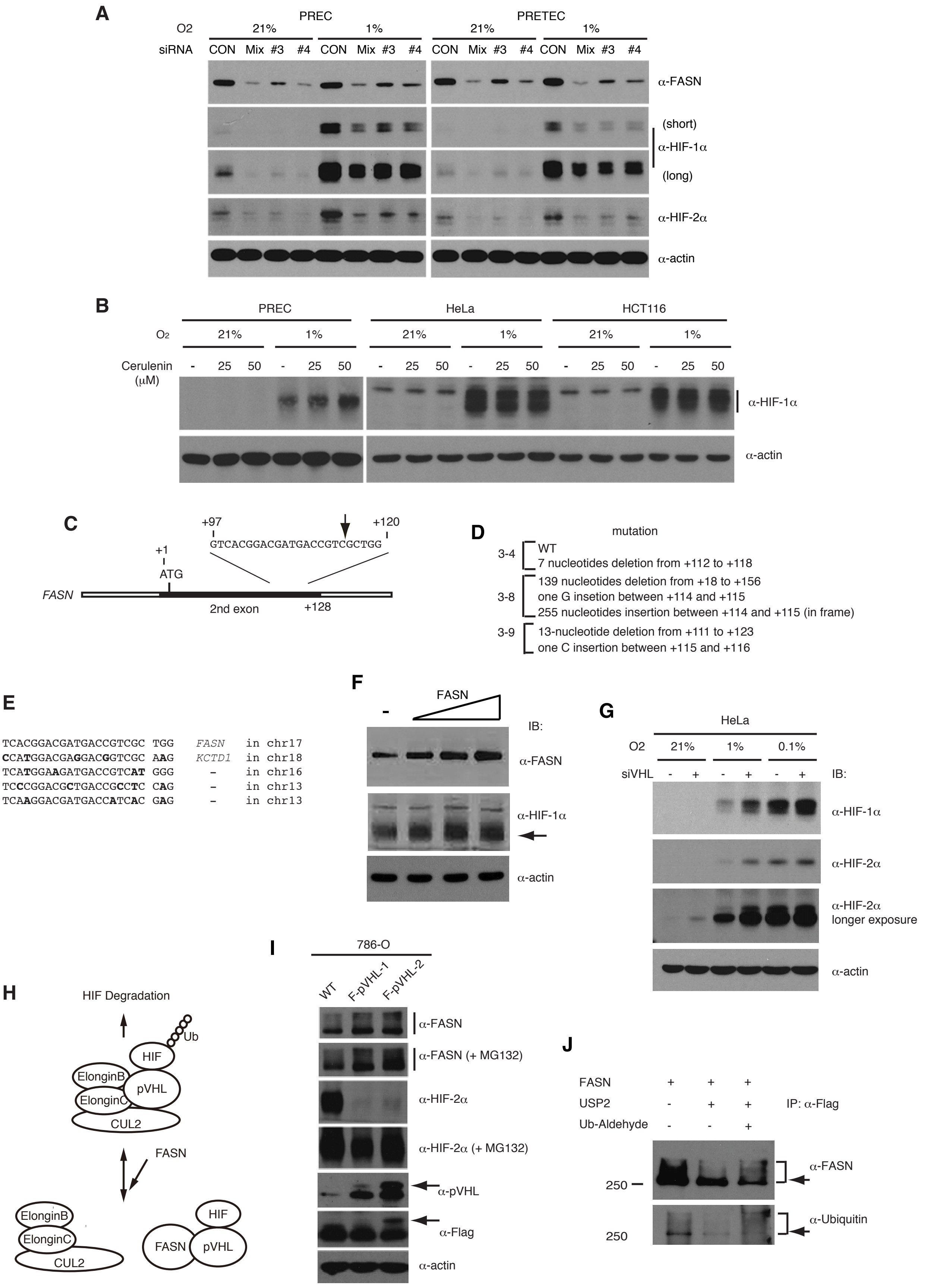

Supplementary Figure 3. FASN regulates HIF-α protein stability. (A) HIF-1α and HIF-2α downregulation by FASN knockdown with pool or individual siRNA in normoxia (21% O_2) and hypoxia (1% O_2). **(B)** Effect of the FASN catalytic inhibitor cerulenin on HIF-1α. PREC, HeLa and HCT116 cells were treated with 0, 25 or 50 μM cerulenin in hypoxia for 4 h, and HIF-1α was detected by western blot analysis. **(C-E)** Alteration of the FASN genomic loci by CRISPR-Cas9. The *FASN*

sequence located at positions 97 to 117 nucleotides downstream of the ATG initiation codon in exon 2 was targeted. Genomic analysis by PCR, cloning and sequencing revealed that clones 3-4, 3-8 and 3-9 had the indicated mutations. Clone 3-4 is heterozygous with one WT allele and 3-8 has one in-frame allele that produces FASN protein with an 85 amino acid deletion that may be responsible for moderate levels of FASN expression. Clone 3-9 contains frame-shift mutations in both alleles and therefore showed almost no FASN expression. A sequence located in the human potassium channel tetramerization domain containing protein 1 gene *KCTD1* and three other sequences located in intergenic loci (-) have been identified using the CRISPR Design web tool (http://crispr.mit.edu) as potential CRISPR-Cas9 off-targeting sites with high scores (0.2). Bold type indicates mismatched nucleotides. By PCR followed by sequencing, it was found that at least one wild type *KCTD1* target sequence was retained in the clones 3-4, 3-8 and 3-9. Intergenic candidate sequences were not examined. **(F)** Effect of FASN overexpression. FASN was transiently expressed in 293T. HIF-1α was slightly enhanced accordingly. **(G)** pVHL was knocked down by siRNA in HeLa cells. Cells were cultured in 21%, 1% or 0.1% oxygen conditions for 4 h and harvested for western blotting. **(H)** Schematic representation of FASN action on the pVHL-containing ubiquitin E3 ligase complex. **(I)** pVHL was stably expressed in 786-O cells using lentiviral vectors (pVHL-1: low expression; pVHL-2: high expression). HIF-2α was efficiently suppressed by pVHL, while the overall levels of FASN proteins were not significantly decreased. This suggested that FASN was not degraded by any pVHL induced modifications. Arrows and lines indicate specific and modified bands respectively. **(J)** The majority of the modified forms of FASN are ubiquitylated. WCEs from HCT116 cells expressing F-pVHL were precipitated and the eluate incubated with the ubiquitin specific peptidase 2 (USP2) in the absence or presence of ubiquitin aldehyde that inhibits de-ubiquitylation. Most of the modified forms of FASN disappeared following incubation with USP2, which could be blocked by ubiquitin aldehyde.

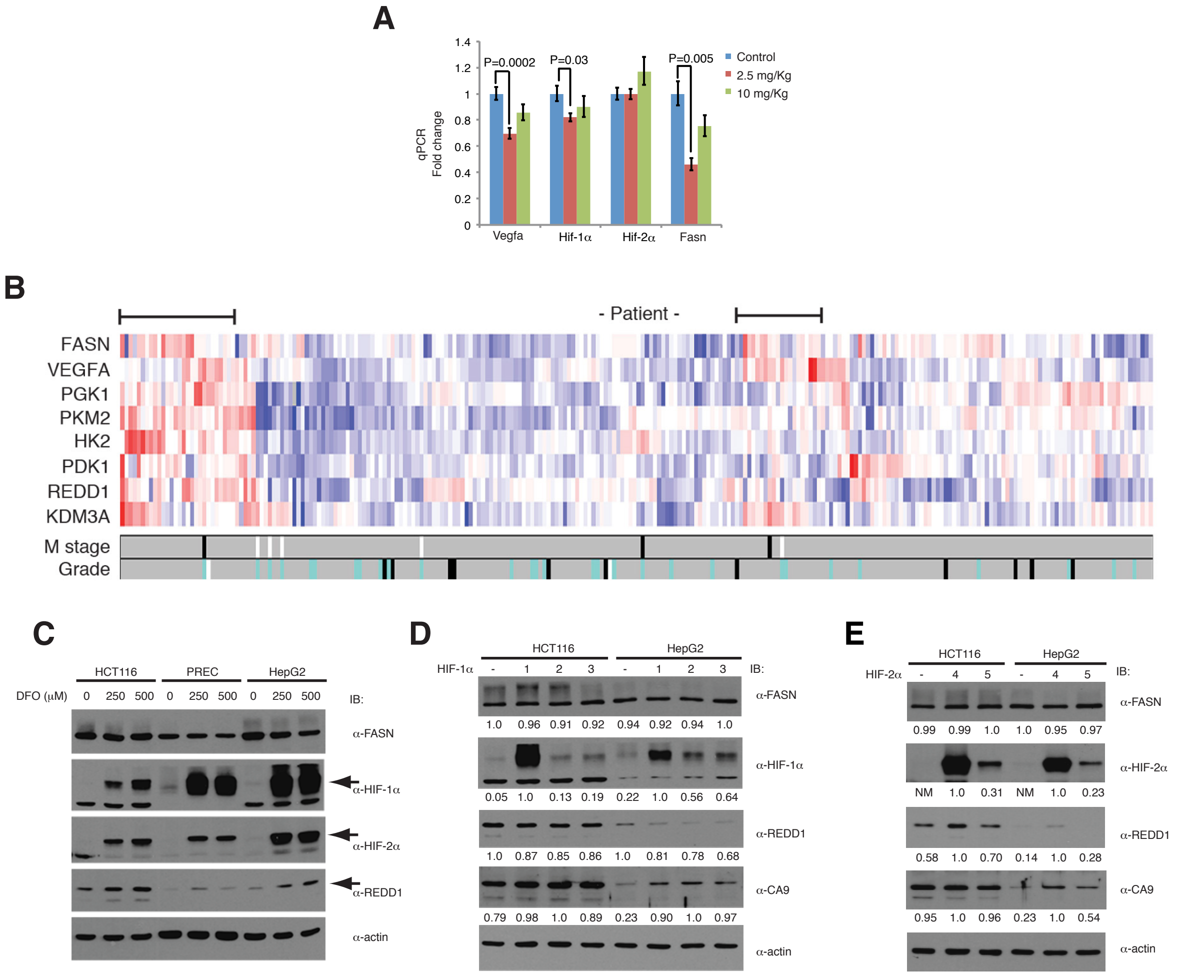

Supplementary Figure 4. Correlation between FASN and HIF target gene expression in gastric cancer and absence of feedback loop between FASN and HIF-α. (A) 25-OH (2.5 mg/Kg or 10 mg/Kg) were injected into the tail vein of mice, and hepatic *Vegfa* and *Fasn* mRNAs levels were analyzed by qPCR. **(B)** Co-overexpression of FASN with HIF target genes in gastric cancer. Gene expression profiles of gastric cancer patients were obtained from IST Online (MediSapiens) as in Fig. 4. See Methods for details. **(C-E)** HIF-α does not repress FASN in cancer cell lines. **(C)** Effects of desferoxamine (DFO) and hypoxia on FASN expression. HCT116, PREC and HepG2 cells were cultured at the indicated concentrations of DFO for 12 h and WCEs were analyzed by western blotting. Effects of HIF-1α **(D)** or HIF-2α **(E)** overexpression on FASN. HIF-1α or HIF-2α was stably expressed in HCT116 or HepG2 using lentiviral vectors (1, by pCSGW or 2 and 3, by CSII-CMV-IRES2-venus) in normoxia. The effects on FASN expression were examined. For band quantification, the highest values are indicated as 1.0.

Supplementary Table 1

Molecular Mass (kDa)	Identity	Calculated molecular mass	The number of Identified peptide		
			Ex1	Ex2	Ex3
250<br>240	gi|41872631|ref|NP_004095.4| fatty acid synthase (FASN)	275874.5	-<br>32	-<br>-	64<br>63
250<br>240	gi|158254191|gb|AAI53882.1| GCN1 general control of amino-acid synthesis 1-like 1	294950.75	-<br>43	-<br>-	27<br>27
250<br>240	gi|18105007|ref|NP_004332.2| CAD protein	245164.84	-<br>26	-<br>-	19<br>15
250<br>240	gi|4505939|ref|NP_000928.1| DNA-directed RNA polymerase II subunit RPB1	218436.03	-<br>-	-<br>-	13<br>1
105<br>95	gi|4504385|ref|NP_001521.1| hypoxia-inducible factor 1-alpha isoform 1	93467.04	-<br>-	7<br>7	-<br>-
105<br>95<br>85<br>75<br>70	gi|19482174|ref|NP_003582.2| cullin-2	87553.98	-<br>0<br>-<br>-<br>-	29<br>19<br>12<br>13<br>4	0<br>0<br>6<br>0<br>0
75	gi|57863257|ref|NP_110379.2| T-complex protein 1 subunit alpha isoform a	60818.86	0	4	9
75	gi|4165018|dbj|BAA37142.1| Acyl-CoA synthetase 3	81263.62	8	0	0
65	gi|14124984|gb|AAH08019.1| Chaperonin containing TCP1, subunit 3 (gamma)	60934.54	20	0	-
	gi|6174877|dbj|BAA86054.1| fatty acid coenzyme A ligase 5	73004.7	9	0	-
	gi|24307939|ref|NP_036205.1| chaperonin containing TCP1, subunit 5 (epsilon)	60089.06	5	2	-
60	gi|57863257|ref|NP_110379.2| T-complex protein 1 isoform a	60818.86	16	6	-
	gi|48762932|ref|NP_006576.2| chaperonin containing TCP1, subunit 8 (theta)	60152.79	14	0	-
	gi|5453607|ref|NP_006420.1| chaperonin containing TCP1, subunit 7 isoform a	59842.19	14	2	-
	gi|5453603|ref|NP_006422.1| chaperonin containing TCP1, subunit 2	57794.33	14	1	-
	gi|4502643|ref|NP_001753.1| chaperonin containing TCP1, subunit 6A isoform a	58443.86	10	3	-
	gi|38455427|ref|NP_006421.2| chaperonin containing TCP1, subunit 4 (delta)	58401.02	10	0	-
	gi|24307939|ref|NP_036205.1| chaperonin containing TCP1, subunit 5 (epsilon)	60089.06	6	1	-
	gi|63162572|ref|NP_005989.3| T-complex protein 1 subunit gamma isoform a	61065.57	0	4	-
25	>gi|4507891|ref|NP_000542.1| von Hippel-Lindau disease tumor suppressor isoform 1	24251.97	-	7	-
25<br>16<br>13	>gi|6005890|ref|NP_009039.1| transcription elongation factor B polypeptide 2 isoform a	13238.58	-	0<br>12<br>6	-
25<br>16<br>13	>gi|5032161|ref|NP_005639.1| transcription elongation factor B polypeptide 1	12636.05	-	12<br>3<br>1	-

Proteins Identified by LC-MS/MS and Number of Identified Peptides. Gel slices of major protein bands visualized by zinc-imidazole staining were analyzed by LC-MS/MS in three independent experiments (Ex 1, 2 and 3). Proteins are listed where more than four peptides appeared in at least one experiment. Bands of molecular weights designated with (-) were not included in the analysis. Common contaminants such as heat shock proteins, tubulins and heterogeneous nuclear ribonucleoproteins were also omitted.

Supplementary Table 2

Gene (human)	
**VEGFA**	forward 5’-AGGAGGAGGGCAGAATCATCA-3’
	reverse 5’-CTCGATTGGATGGCAGTAGCT-3’
**PGK1**	forward 5’-AGATTCAGCTAGTGGCCAAGAG-3’
	reverse 5’-TGCAGTGAAGATGAGCTGAGAT-3’
**GLUT1/SLC2A1**	forward 5’-GGAGCCCAGCAGCAAGAAG-3’
	reverse 5’-ACTGCAGGGAGCCAAGCA-3’
**ADM**	forward 5’-CCCTGATGTACCTGGGTTCG-3’
	reverse 5’-GCCCACTTATTCCACTTCTTTCG-3’
**CA9**	forward 5’-TGCCTATGAGCAGTTGCTGTCT-3’
	reverse 5’-CAGTCCTGGGACCTGAGTCTCT-3’
**KDM3A**	forward 5’-ACTGCTTCTGGCTTACTCAGG-3’
	reverse 5’-TCACTGGCTTTCAGCATAGCA-3’
**MXI1**	forward 5’-GATTCAGAGCGAGAGGAGATTG-3’
	reverse 5’-ACACTGGCACTGGAGTAACC-3’
**REDD1/DDIT4**	forward 5’-GAACTCCCACCCCAGATCGG-3’
	reverse 5’-CGAGGGTCAGCTGGAAGGTG-3’
**HK2**	forward 5’-GATTGTCCGTAACATTCTCATCGA-3’
	reverse 5’-TGTCTTGAGCCGCTCTGAGAT-3’
**PDK1**	forward 5’-ATTCAAGTTCATGTCACGCTGG-3’
	reverse 5’-TTTCCTCAAAGGAACGCCAC-3’
**PKM2**	forward 5’-GCCTGCTGTGTCGGAGAAG-3’
	reverse 5’-CAGATGCCTTGCGGATGAATG-3’
**HIF-1α**	forward 5’-TTCCAGTTACGTTCCTTCGATC-3’
	reverse 5’-TTTGAGGACTTGCGCTTTCA-3’
**HIF-2α**	forward 5’-GTGCTCCCACGGCCTGTA-3’
	reverse 5’-TTGTCACACCTATGGCATATCA-3’
**FASN**	forward 5’-CATCCAGATAGGCCTCATAGAC-3’
	reverse 5’-CTCCATGAAGTAGGAGTGGAAG-3’
**PPIA**	forward 5’-ACGGCGAGCCCTTGG-3’
	reverse 5’-TTTCTGCTGTCTTTGGGACCT-3’

Gene (mouse)	
**Vegfa**	forward 5’-TACCTCCACCATGCCAAGTG-3’
	reverse 5’-TCATGGGACTTCTGCTCTCCTT-3’
**Pgk1**	forward 5’-TATCTTGGGAGGCGCTAAAGTT-3’
	reverse 5’-ATCTCCATGTTGTTGAGCACCT-3’
**Redd1**	forward 5’-TGGTGCCCACCTTTCAGTTG-3’
	reverse 5’-GTCAGGGACTGGCTGTAACC-3’
**Pdk1**	forward 5’-CTGGCACTGCCTTCTACTCA-3’
	reverse 5’-ATGCCGCTGTAACGGTGAA-3’
**Pkm2**	forward 5’-TGTCTGGAGAAACAGCCAAG-3’
	reverse 5’-TCCTCGAATAGCTGCAAGTG-3’
**Hif-1α**	forward 5’-TCATCAGTTGCCACTTCCCCAC-3’
	reverse 5’-CCGTCATCTGTTAGCACCATCAC-3’
**Hif-2α**	forward 5’-CCAGTTCCAGGACTACGGTC-3’
	reverse 5’-CAGTTCCGGCAACAGGTAAG-3’
**Fasn**	forward 5’-GAGGACACTCAAGTGGCTGA-3’
	reverse 5’-GTGAGGTTGCTGTCGTCTGT-3’
**Ppia**	forward 5’-CTGCACTGCCAAGACTGAATG-3’
	reverse 5’-CACAATGTTCATGCCTTCTTTCAC-3’
**Tbp**	forward 5’-ACCCTTCACCAATGACTCCTATG-3’
	reverse 5’-TGACTGCAGCAAATCGCTTGG-3’

Primer sequences used for analysis of human and mouse genes by qPCR are listed.
